# Dual-Energy X-Ray Absorptiometry Derived Adiposity Measures and Pre-Frailty/Frailty among Norwegian Adults: The Tromsø Study 2007–2015

**DOI:** 10.1007/s12603-023-1920-2

**Published:** 2023-05-25

**Authors:** Shreeshti Uchai, L.F. Andersen, J. Johansson, L.A. Hopstock, A. Hjartåker

**Affiliations:** 1 Department of Nutrition, Institute of Basic Medical Sciences, University of Oslo, Norway, Postbox: 1046, Blindern, 0317, Oslo, Norway; 2 Department of Community Medicine, UiT The Arctic University of Norway, Tromsø, Norway

**Keywords:** DXA, fat mass, frailty, pre-frailty, VAT, visceral fat

## Abstract

**Objectives:**

Aging is associated with changes in body composition. Excess adiposity among older adults has been linked with metabolic syndromes and aggravated age-associated decline in physical functioning. Few longitudinal studies have explored the association between dual-energy X-ray absorptiometry (DXA)-derived total as well as central adiposity measures and frailty. We examined the association of DXA-derived total and central adiposity with pre-frailty/frailty among Norwegian adults after 8 years of follow-up.

**Design:**

Prospective observational study.

**Setting:**

Community-dwelling adults from Tromsø, Norway.

**Measurements:**

Adiposity was defined by fat mass index (FMI) and visceral adipose tissue (VAT) mass assessed using DXA measures. Frailty status was assessed by low grip strength, slow walking speed, exhaustion, unintentional weight loss and low physical activity level. Pre-frail and frail participants at baseline were excluded. Sex-stratified multivariable logistic regression models were used to investigate the association.

**Results:**

Participants comprised 234 women (mean age 68 years) and 146 men (mean age 69 years) attending the population-based Tromsø Study in 2007–2008 (Tromsø6) and 2015–2016 (Tromsø7). At the end of follow-up, 25.6% of the women and 27.4% of the men were pre-frail/frail. Compared with women in the lowest tertiles, those in the highest tertile of baseline FMI (odds ratio [OR] 4.42, 95% confidence interval [CI] 1.88–10.35) and VAT mass (OR 2.47, 95% CI 1.10–5.50), respectively had higher odds for pre-frailty/frailty at follow-up.

**Conclusion:**

We found a higher likelihood of pre-frailty/frailty in later years among women with general and central adiposity in adulthood, highlighting the importance of preventing excess adiposity for healthy aging.

## Introduction

Globally, the population is aging rapidly (1) and geriatric syndromes such as frailty are on the rise. Frailty increases the vulnerability of older adults to falls, fractures, disability, hospitalization, reduced quality of life and mortality (2, 3). Frailty is characterized by decreased resistance to external stressors resulting from cumulative decline across several physiologic systems (4). According to Fried et al., physical frailty is identified as the presence of three or more, and pre-frailty as the presence of one or two, of the given five criteria: unintentional weight loss, self-reported exhaustion, weakness, slow walking speed and low physical activity (4). Pre-frailty is a multi-factorial, prodromal risk state, often predisposing to and preceding frailty (5, 6). Identifying pre-frailty provides an opportunity to prevent, delay or reverse frailty and the associated adverse outcomes (5, 6). The prevalence of physical frailty and pre-frailty was estimated to be 12% and 46%, respectively, among community-dwelling adults aged ≥50 years by a recent systematic review that included population-based studies from 62 countries (7).

Aging is often accompanied by changes in body composition, such as a decrease in muscle mass, an increase in fat mass and redistribution of fat mass from the subcutaneous to the abdominal visceral compartment (8, 9, 10, 11). Fat mass represents total, whole-body adiposity and visceral adipose tissue (VAT) mass represents the metabolically active fat in the abdominal region. Body mass index (BMI) and waist circumference (WC) have been widely used as indirect measures of total and visceral fat mass, respectively. Despite their effectiveness in assessing adiposity-related risks at the population level (12, 13), BMI does not effectively distinguish between fat and muscle mass (13) and WC does not distinguish between visceral and subcutaneous fat (14). Thus, these anthropometric measures might not properly capture underlying age-associated changes in fat mass content and redistribution (15, 16). Therefore, adiposity measures assessed using advanced device-based methods of body composition analysis such as dual-energy X-ray absorptiometry (DXA), computed tomography (CT) and magnetic resonance imaging (MRI) are preferred for higher accuracy.

Excess adiposity, especially visceral fat, is linked to inflammation, oxidative stress and various metabolic syndromes correlated with the risk of frailty (10, 17). Various studies have observed a positive association of total and central adiposity, assessed by traditional anthropometric measures such as BMI (18, 19, 20, 21, 22) and WC (23, 24, 25, 26, 27), with frailty. Fewer studies have assessed the association between fat mass and frailty risk, finding no significant association (28, 29) or positive association (23, 24, 26, 30, 31). Out of these, the number of longitudinal studies exploring the association between DXA-derived fat mass measures and frailty for longer follow-up periods are limited (31). With regards to the association between VAT mass and frailty, we found three studies reporting positive associations (32, 33, 34). All three have cross-sectional designs, making it challenging to establish the directionality of the association and two of these studies (33, 34) have used bioelectrical impedance analysis (BIA) derived VAT measures which have limited accuracy.

Therefore, with the aim to strengthen the evidence base, we investigated the longitudinal association between DXA-derived baseline total and central adiposity and the risk of pre-frailty/frailty among community-dwelling Norwegian adults after 8 years of follow-up.

## Methods

### Study sample

The present study included participants from the population-based Tromsø Study. It consists of seven surveys: Tromsø1 (1974), Tromsø2 (1979–1980), Tromsø3 (1986–1987), Tromsø4 (1994–1995), Tromsø5 (2001), Tromsø6 (2007–2008) and Tromsø7 (2015–2016), inviting total birth cohorts and random samples registered as inhabitants in the Tromsø municipality, Norway. Starting from Tromsø4, random and selected samples have been invited to a second visit, approximately 14 days after the first visit, for various additional clinical examinations including DXA-derived adiposity measures.

Since the earlier surveys did not have detailed information on VAT measures, the present study uses data from Tromsø6 (baseline) (35) and Tromsø7 (follow-up) (36). The follow-up duration was 8 years. Tromsø6 included 12,977 participants aged 30–87 years and Tromsø7 included 21,083 participants aged 40–99 years. The information on selection, recruitment and attendance of the participants in Tromsø6 and Tromsø7 have been discussed in detail elsewhere (35, 36). In total, 906 participants from Tromsø6 and 3670 participants from Tromsø7 underwent whole-body DXA scans.

**Figure 1:**
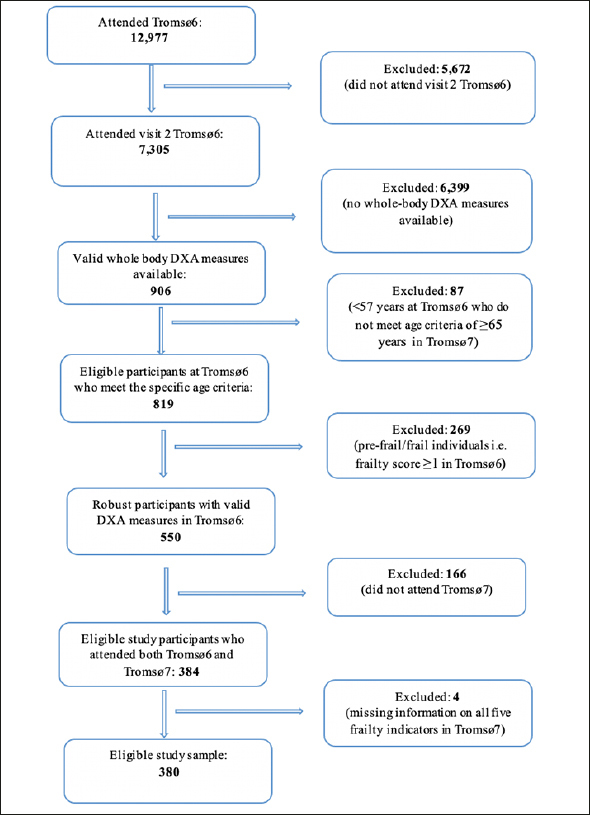
Flowchart displaying participants' inclusion and exclusion

We included participants with valid whole-body DXA scans at Tromsø6 who attended Tromsø7. We excluded those younger than 65 years in Tromsø7 (Tromsø6 <57 years) (n = 87), those who were pre-frail/frail (frailty score ≥1) in Tromsø6 (n = 269) and those with missing information on all five frailty indicators in Tromsø7 (n = 4), leaving 380 participants in Tromsø6 for primary analysis (Figure 1).

### Ethics

The Tromsø Study is approved by the Regional Committee of Medical and Health Research Ethics (REK) North and the Norwegian Data Protection Authority. All participants in Tromsø6 and Tromsø7 provided written informed consent. Approvals from REK (ref. 2021/234146) and the Norwegian Centre for Research Data (NSD; ref. 364331) were obtained for the present study.

### Body composition measures

BMI was calculated as body weight in kilograms divided by the square of body height in meters (kg/m^2^), measured in light clothes with no footwear. WC was measured to the nearest centimeter at the umbilicus level. Trained technicians performed DXA scans in line with the manufacturer's protocols, using a Lunar Prodigy Advance (GE Medical Systems, Madison, Wisconsin, USA) device in Tromsø6 and Tromsø7. Each morning before the measurements, the DXA device was calibrated using a phantom. Post-scan images were inspected and relevant quality corrections were made according to a standardized protocol.

Total body fat mass in grams was directly obtained from the DXA measurement. The fat mass index (FMI; kg/m^2^) was calculated by dividing fat mass in kilograms by the square of height in meters. FMI eliminates the confounding from height and has been reported to be a more accurate screening tool for predicting metabolic syndromes (37) compared with the more commonly used body fat percentage. The validated CoreScan application (EnCore version 17.0, GE Healthcare, Madison, Wisconsin, USA) (38) was used to compute VAT mass in grams and volume in cm^3^ from existing DXA scans. As perfect correlation (r = 1) between VAT mass and VAT volume was observed among both women and men, VAT mass was selected for further analysis to harmonize with the other body composition parameters. In our primary analytic sample, two participants had VAT mass values equal to 0 and were manually transformed into the lowest registered value of VAT mass in the Tromsø6 sample (i.e., 2 g) in accordance with Lundblad et al. (39).

As body composition varies among women and men (40), FMI and VAT mass were categorized into sex-specific tertiles. FMI was categorized among women as low (first tertile: <8 kg/m^2^), medium (second tertile: 8–10.7 kg/m^2^) and high (third tertile: >10.7 kg/m^2^) and among men as low (first tertile: <5.7 kg/m^2^), medium (second tertile: 5.7–7.3 kg/m^2^) and high (third tertile: >7.3 kg/m^2^). VAT was categorized among women as low (first tertile: <500 g), medium (second tertile: 500–990 g) and high (third tertile: >990 g) and among men as low (first tertile: <1047 g), medium (second tertile: 1047–1674 g) and high (third tertile: >1674 g).

### Frailty assessment

We assessed three self-reported measures (unintentional weight loss, exhaustion and low physical activity) and two performance-based measures (walking speed and low grip strength) to operationalize physical frailty, in line with Fried et al.'s frailty phenotype definition (4). All indicators were assessed at both time points, except for walking speed (missing in Tromsø6).

In Tromsø6 and Tromsø7, self-reported involuntary weight loss during the last 6 months was measured using the Malnutrition Universal Screening Tool (41), exhaustion was defined by the response “pretty much” or “very much” to the question “During the last week, have you experienced that everything is a struggle?” from the Hopkins' Symptom Checklist-10 (42), low physical activity level was defined by the response “Reading, watching TV/screen or other sedentary activity” to the question “Describe your exercise and physical exertion in leisure time over the last year” from the Saltin-Grimby Physical Activity Level Scale (43) and weakness was defined by sex- and BMI-specific cut-offs for grip strength as suggested by Fried et al. (4). Grip strength (kg) was measured in Tromsø7 using a calibrated Jamar+ Digital Dynamometer (notch 2) (Patterson Medical, Warrenville, IL, USA), following the Southampton protocol procedures (44), and in Tromsø6 (bar) using a Martin vigorimeter (balloon sizes 3 and 5 for women and men, respectively). The grip strength measures in Tromsø6 and Tromsø7 were made comparable using validated conversion factors (45). The grip strength measure (bar) in Tromsø6 was multiplied by 100 to convert it into kilopascals (kPa). Then the measure in kPa was divided by the conversion factor obtained from the Jamar–Martin conversion table. The conversion factor used for females to convert Martin (balloon 3) to Jamar (notch 2) was 2.43 and for males to convert Martin (balloon 5) to Jamar (notch 2) was 1.68 (45). Walking speed at Tromsø7 was measured according to the Short Physical Performance Battery (SPPB) protocol (46), where the fastest time out of two walks was selected and converted to seconds per 15 feet from seconds per 4 meters, with sex- and height-adjusted cut-offs, according to Fried et al. (4).

Participants were classified as robust (0), pre-frail (1–2) and frail (≥3) based on the number of frailty indicators. As the frailty prevalence was low, we combined pre-frail and frail individuals to form a common outcome, i.e., pre-frail/frail (frailty score ≥1) at Tromsø7. We have compared each frailty indicator assessed in our study in Tromsø6 and Tromsø7 with Fried et al.'s definition in detail (Supplementary Table 1).

### Covariates

Sociodemographic characteristics included age, sex, education (primary/partly secondary education [schooling up to 10 years], upper secondary education [minimum of 3 years] and college/university) and marital/cohabitation status. Self-reported lifestyle factors included smoking (current, former or never smoker), alcohol intake (never-drinker, infrequent drinker [<2–4 times/month] and frequent drinker [>2–3 times/week]) and social support (not enough good friends or enough good friends). Self-perceived health status was categorized as poor or good (47). Charlson's comorbidity index (48) was used to assess comorbidity (without weighting the diseases) if prevalent self-reported coronary heart disease (angina pectoris/myocardial infarction), stroke, diabetes, hypertension, kidney disease, pulmonary disease (asthma/chronic bronchitis/emphysema), osteoporosis and/or peptic ulcer was present.

### Statistical analysis

The sociodemographic and lifestyle factors, as well as body composition measures at baseline across robust and pre-frail/frail groups, are described, using proportion and count for categorical variables and mean and standard deviation (SD), or median and interquartile range, for continuous variables depending on their distribution. Differences between robust and pre-frail/frail groups were tested using the χ2 test for categorical variables and Student's t-test or Mann–Whitney U-test for continuous variables.

The association between baseline adiposity (Tromsø6) and pre-frailty/frailty at follow-up (Tromsø7) was assessed using multiple logistic regression models reporting odds ratios (ORs) and 95% confidence intervals (CIs). Separate longitudinal models were fitted for total adiposity (FMI) and central adiposity (VAT), and all the analyses were stratified by sex due to significant differences in body composition (40). The adiposity measures were entered in the model separately in continuous as well as categorical form (tertiles). All the models were minimally adjusted for age and further adjusted for educational level, marital/cohabitation status, social support, alcohol intake, smoking status and self-perceived health at baseline. Variation inflation factor (VIF) values were used to test multicollinearity in the model, with values <5 considered acceptable.

In supplementary analyses, we assessed the association of adiposity measures with each frailty component. Furthermore, the baseline characteristics of eligible study participants from Trom06 were compared with otherwise eligible participants from Trom06 who were lost to follow-up.

All the statistical analyses were performed using STATA 16 (49). Statistical significance was set at P <0.05.

## Results

### Sociodemographic and lifestyle factors of the study population

Mean baseline age was 67.7 years in women, who constituted 62% of our study sample, and 69 years in men. Table 1 displays the sex-stratified baseline characteristics of the study population by frailty status at follow-up, of which 25.6% of women (1.3% frail) and 27.4% of men (0.7% frail) were pre-frail/frail. The mean age of pre-frail/frail women at follow-up was 75.8 years, whereas that of pre-frail/frail men was 78.4 years. A significantly higher proportion of women (P = 0.05) and men (P = 0.01) who perceived their health to be good at baseline remained robust at follow-up. There was no significant difference between baseline marital/cohabitation status, social support and the development of pre-frailty/frailty.

**Table 1:** Sex-stratified baseline characteristics of participants by frailty status: The Tromsø Study 2007–2015

	**Women (n = 234)**				**Men (n = 146)**		
	**Frailty status**		**P value**	**Frailty status**			**P value**
	**Robust % (n) 74.4 (174)**	**Prefrail/frail % (n) 25.6 (60)**		**Robust % (n) 72.6 (106)**		**Prefrail/frail % (n) 27.4 (40)**	
Age, mean (SD)	67.6 (5.0)	67.8 (4.4)	0.76^a^	Age, mean (SD)	68.5 (5.9)	70.4 (5.2)	0.09^a^
Married/Cohabiting	73.0 (127)	70.0 (42)	0.66	Married/Cohabiting	84.0 (89)	87.5 (35)	0.59
Self-perceived health status - good	71.7 (124)	58.3 (35)	0.05	Self-perceived health status - good	76.7 (79)	55.0 (22)	0.01
Social support - enough good friends	94.7 (160)	89.7 (52)	0.18	Social support - enough good friends	89.8 (88)	88.9 (32)	0.88
Educational level				Educational level			
Primary/Partly secondary	43.6 (75)	52.5 (31)		Primary/Partly secondary	28.3 (30)	22.5 (9)	
Upper secondary	29.7 (51)	27.1 (16)	0.45	Upper secondary	34.9 (37)	37.5 (15)	0.78
College/University	26.7 (46)	20.4 (12)		College/University	36.8 (39)	40.0 (16)	
Smoking status				Smoking status			
Current smoker	12.7 (22)	16.7 (10)		Current smoker	7.6 (8)	17.5 (7)	
Former smoker	42.2 (73)	40.0 (24)	0.75	Former smoker	64.2 (68)	62.5 (25)	0.17
Never	45.1 (78)	43.3 (26)		Never	28.3 (30)	20 (8)	
Alcohol				Alcohol			
Never/Abstaining	15.3 (26)	27.1 (16)		Never/Abstaining	11.4 (12)	5.0 (2)	
Infrequent drinkers	60.0 (102)	55.9 (33)	0.09	Infrequent drinkers	66.7 (70)	77.5 (31)	0.37
Frequent drinkers	24.7 (42)	17.0 (10)		Frequent drinkers	21.9 (23)	17.5 (7)	
Comorbidity	20.1 (35)	25.0 (15)	0.43	Comorbidity	27.4 (29)	35.0 (14)	0.37

Values are percentages (numbers) except when mentioned otherwise. P value: χ2 test for categorical variables. ^a^P value: Student's t-test. SD, standard deviation.

### Body composition measures

Table 2 describes the baseline body composition measures of the study population by frailty status at follow-up. Mean BMI (P <0.01) and WC (P <0.01) at baseline were higher among women pre-frail/frail women at follow-up compared with robust women, whereas there was no significant difference in mean baseline BMI (P = 0.39) or WC (P = 0.54) among pre-frail/frail versus robust men. Similarly, mean baseline FMI (10.8 kg/m^2^ versus 9.1 kg/m^2^) and VAT mass (995 g versus 789 g) were significantly higher among women classified as pre-frail/frail at follow-up than robust women. The average baseline FMI (7.1 kg/m^2^ versus 6.6 kg/m^2^) and VAT mass (1580 g versus 1413 g) were higher in pre-frail/frail men at follow-up compared with robust men; however, the difference was not statistically significant (P = 0.25). A significantly higher proportion of women in the highest tertile of FMI and highest tertile of VAT mass at baseline was classified as pre-frail/frail at follow-up, whereas among men there were no significant differences between the proportions of men in different tertiles of FMI and VAT mass who were classified as robust or pre-frail/frail.

**Table 2:** Sex-stratified baseline body composition measures of participants by frailty status: The Tromsø Study 2007–2015

	**Women (n = 234)**				**Men (n = 146)**		
	**Frailty status**		**P value**		**Frailty status**		**P value**
	**Robust % (n) 74.4 (174)**	**Prefrail/frail % (n) 25.6 (60)**		**Robust % (n) 72.6 (106)**		**Prefrail/frail % (n) 27.4 (40)**	
BMI (kg/m^2^)	25.8 (4.2)	27.9 (3.6)	< 0.01^a^	BMI (kg/m^2^)	26.8 (2.8)	27.3 (3.8)	0.39^a^
WC (cm)	88.6 (10.7)	96.3 (10.3)	< 0.01	WC (cm)	99.3 (8.4)	100.3 (9.7)	0.54
FMI (kg/m^2^)	9.1 (3.1)	10.8 (2.8)	< 0.01	FMI (kg/m^2^)	6.6 (2.1)	7.1 (2.7)	0.25
FMI tertiles				FMI tertiles			
Low (T1)	37.0 (66)	20.0 (12)	< 0.01	Low (T1)	34.0 (36)	32.5 (13)	0.95
Medium (T2)	35.1 (61)	28.3 (17)		Medium (T2)	34.0 (36)	32.5 (13)	
High (T3)	27.0 (47)	52.7 (31)		High (T3)	32.0 (34)	35.0 (14)	
VAT (g)	789 (546)	995 (612)	0.01	VAT (g), mean (SD)	1413 (712)	1580 (881)	
Median (Q1-Q3)	635 (385–1057)	828 (529–1393)	0.01^b^	Median (Q1–Q3)	1332(915–1853)	1537 (1015–1973)	0.33^b^
VAT tertiles				VAT tertiles			
Low (T1)	36.8 (64)	23.3 (14)	0.09	Low (T1)	35.9 (38)	27.5 (11)	0.53
Medium (T2)	33.3 (58)	33.3 (20)		Medium (T2)	31.1 (33)	40.0 (16)	
High (T3)	29.9 (52)	43.4 (26)		High (T3)	33.0 (35)	32.5 (13)	

Values are mean values (standard deviations) or percentages (numbers). P value: χ2 test for categorical variables. P value: ^a^Student's t-test; ^b^Mann-Whitney U-test. BMI, body mass index; FMI, fat mass index; SD, standard deviation; VAT, visceral adipose tissue; WC, Waist circumference. Q1: first quartile (25^th^ percentile); Q3: third quartile (75^th^ percentile). T1: first tertile; T2: second tertile; T3: third tertile.

FMI categories

Low (T1): women <8 kg/m^2^; men <5.7 kg/m^2^

Medium (T2): women 8.0–10.7 kg/m^2^; men 5.7–7.3 kg/m^2^

High (T3): women >10.7 kg/m^2^; men >7.3 kg/m^2^

VAT categories

Low (T1): women <500 g; men <1047 g

Medium (T2): women 500–900 g; men 1047–1674 g

High (T3): women >990 g; men >1674 g

### Adiposity measures and pre-frailty/frailty

Table 3 displays the sex-stratified longitudinal association between the adiposity measures at baseline and pre-frailty/frailty at follow-up. When adjusted for potential covariates, with every 1-kg/m^2^ increase in baseline FMI, the odds of pre-frailty/frailty significantly increased by 18% among women, whereas, among men, there was a non-significant increase of 11%. Similarly, with every 100-g increase in baseline VAT mass, the odds of pre-frailty/frailty significantly increased by 7% among women, whereas, among men, there was a weaker non-significant increase of 2%.

**Table 3:** Sex-stratified longitudinal association between DXA-derived adiposity measures pre-frailty/frailty: The Tromsø Study 2007–2015

	**Women (n = 234)**				**Men (n = 146)**		
	**Model 1 OR (95% CI)**	**Model 2 OR (95% CI)**	**P trend**		**Model 1 OR (95%CI)**	**Model 2 OR (95%CI)**	**P trend**
FMI (kg/m^2^)	1.20 (1.08–1.32)	1.18 (1.06–1.32)		FMI (kg/m^2^)	1.11 (0.95–1.31)	1.11 (0.92–1.33)	
FMI tertiles				FMI tertiles			
Low (T1)	Ref	Ref	<0.01	Low (T1)	Ref	Ref	0.53
Medium (T2)	1.53 (0.68–3.48)	1.52 (0.64–3.64)		Medium (T2)	1.00 (0.40–2.48)	1.42 (0.49–4.15)	
High (T3)	3.63 (1.68–7.82)	4.42 (1.88–10.35)		High (T3)	1.24 (0.50–3.05)	1.41 (0.49–4.04)	
VAT (g)	1.06 (1.01–1.12) ^a^	1.07 (1.01–1.13) ^a^		VAT(g)	1.03 (0.98–1.08)^a^	1.02 (0.97–1.08) ^a^	
VAT tertiles				VAT tertiles			
Low (T1)	Ref	Ref	0.03	Low (T1)	Ref	Ref	0.70
Medium (T2)	1.57 (0.73–3.40)	1.53 (0.67–3.45)		Medium (T2)	1.54 (0.62–3.84)	1.82 (0.64–5.13)	
High (T3)	2.28 (1.08–4.81)	2.47 (1.10–5.50)		High (T3)	1.34 (0.53–3.40)	1.23 (0.42–3.62)	

Model 1: adjusted for age. Model 2: adjusted for age, educational level, marital/cohabitation status, social support, alcohol intake, smoking status and self-perceived health at baseline. ^a^ORs (95% CI) are per 100-g increase in the VAT mass. P value: test for linear trend. CI, confidence interval; FMI, fat mass index; OR, odds ratio; VAT, visceral adipose tissue. T1, first tertile; T2, second tertile; T3, third tertile.

FMI categories

Low (T1): women <8 kg/m^2^; men <5.7 kg/m^2^

Medium (T2): women 8.0–10.7 kg/m^2^; men 5.7–7.3 kg/m^2^

High (T3): women >10.7 kg/m^2^; men >7.3 kg/m^2^

VAT categories

Low (T1): women <500 g; men <1047 g

Medium (T2): women 500–900 g; men 1047–1674 g

High (T3): women >990 g; men >1674 g

Among women, there was a significant trend of becoming pre-frail/frail with higher FMI and VAT mass. Compared with the lowest FMI tertile, women in the highest tertile at baseline had 4.42 times higher odds of being pre-frail/frail at follow-up (OR 4.42, 95% CI 1.88–10.35) in a fully adjusted model. Similarly, when compared with the lowest tertile of baseline VAT mass, women in the highest tertile had 2.47 times higher odds of pre-frailty/frailty (OR 2.47, 95% CI 1.10–5.50).

Compared with men in the lowest tertile at baseline, those in the medium FMI tertile followed by the highest FMI tertile and those in medium VAT tertile followed by the highest VAT tertile were observed to have elevated odds of being pre-frail/frail at follow-up, when adjusted for potential covariates, but none of the associations was statistically significant for men and there was no significant trend.

In a smaller sub-sample of participants with information available on cognitive function, we further adjusted for the cognitive status in our model and found no significant changes in our findings (data not shown). In addition, we assessed the association of baseline FMI and VAT with each frailty indicator at follow-up using minimally adjusted models (Supplementary Table 2). Among women, FMI was significantly associated with low walking speed and low physical activity level, whereas VAT mass was significantly associated with low physical activity level only. Among men, FMI was significantly associated with low walking speed and low physical activity level, whereas VAT mass was significantly associated with low walking speed only. However, the number of men with low grip strength (n = 5) and exhaustion (n = 3) was very low in the present study.

When compared with the eligible study participants from Tromsø6 who attended Tromsø7, those lost to follow-up were older and had slightly higher BMI, WC, FMI and VAT mass, and also varied somewhat with regard to other risk factors for pre-frailty and frailty (Supplementary Table 3).

## Discussion

The present study followed 380 women and men from Tromsø6 for 8 years and examined the association between DXA-derived adiposity measures at baseline and the odds of developing pre-frailty/frailty at follow-up. The prevalence of frailty and pre-frailty was 1.3% and 24.4% among women and 0.7% and 26.7% among men, respectively. We observed a significant positive association between DXA-derived adiposity measures, i.e., FMI and VAT mass, and odds of pre-frailty/frailty in women, whereas, in men, we detected a weak positive association close to unity. Women in the highest tertile of baseline FMI or VAT mass had a pronounced likelihood of becoming pre-frail/frail later in life.

Excess adiposity, assessed with anthropometric measures such as BMI or WC, has been observed to be associated with an increased risk of frailty in previous cross-sectional (19, 23, 24, 25, 30) as well as longitudinal (21, 22, 27) studies. However, longitudinal evidence about the association between excess adiposity, assessed with device-based advanced body composition methods, and frailty seems to be limited and inconclusive. Some cross-sectional studies have reported frail individuals as being characterized by higher body fat mass percentage (23, 24, 26, 30), whereas others have reported no significant association between frailty and body fat mass percentage (28) or FMI (29). Our finding of a positive association between fat mass and pre-frailty/frailty in women contrasts with a cross-sectional study from Tanaka et al. (50), who observed a significant positive association between body fat percentage and pre-frailty/frailty in older men (n = 84, mean age 75.5 years) but not in older women (n = 249, mean age 72.5 years) (50). Hirani et al. (31) reported a significant positive association between body fat percentage and the risk of frailty in a longitudinal study among older men (age ≥70 years). The use of body fat percentage to assess adiposity, which cannot be evaluated independently from lean mass, might explain this difference to a certain extent. Only a few studies have investigated the association between VAT and frailty among middle-aged and older adults. All of them have cross-sectional designs and differ from our study in terms of the VAT assessment methods. A study conducted among community-dwelling older adults (n = 214, mean age 75.4 years, 69.7% women) reported a positive association between visceral fat area (VFA) measured using BIA and pre-frailty (34). Another study conducted among middle-aged and older adults (n = 483, mean age ≥45 years) reported a positive association between BIA-derived VFA and frailty but only among women (n = 188) (33), which aligns with our findings. Although BIA provides acceptable estimates for fat mass, its accuracy for estimating VAT is severely limited compared with DXA (51). On the contrary, a positive association between increased VAT area (assessed using CT) and frailty was observed among adult men with and without human immunodeficiency virus (n = 399, mean age 60 years) by Hawkins et al. (32).

Falsarella et al. examined the cross-sectional association of fat mass with each frailty indicator individually and reported a significant association of fat percentage with slow walking speed, low physical activity and low grip strength (26). Increased body fat was found to be associated with poor physical performance assessed using the SPPB regardless of sex by Kim et al. (52), whereas two other studies reported this association to be significant only among women (29, 53). In the present study, baseline FMI was associated with slow walking speed and low physical activity at follow-up in both women and men, whereas baseline VAT was observed to be associated with low physical activity among women and slow walking speed among men. Previous cross-sectional studies have found significant associations between VFA and self-reported slow walking speed among older adults (34), as well as correlations for the VFA, adjusted for total fat, and walking speed and grip strength among older men but not among older women (33). In our study, baseline FMI and VAT mass were not significantly associated with unintentional weight loss, exhaustion or low grip strength among both sexes. However, there were very few men with low grip strength and exhaustion.

There might be multiple underlying mechanisms behind the association between increased adiposity and the development of frailty among older adults. Aging is accompanied by changes in sex hormones, cellular composition and basal metabolic rate (10). Combined with various unhealthy lifestyle factors, these might potentially result in decreasing muscle mass, increasing fat mass and redistribution of fat to the abdominal visceral compartment (8, 9, 10, 11). Aging is also characterized by chronic low-grade inflammation (10). Excess fat, especially visceral fat mass, aggravates this age-associated inflammation and oxidative stress further and contributes to the increased accumulation of various health deficits affecting physical functionality and increasing the risk of frailty (10, 54, 55, 56). Furthermore, excess adiposity is associated with lipid accumulation in muscle fibres, resulting in impaired muscle quality, strength and physical functioning, contributing to frailty (54, 57). However, all these mechanisms should be interpreted in the light of sexual dimorphism in body composition. Men generally have lower body fat percentages than premenopausal women but tend to deposit more adipose tissue in the abdominal visceral compartments (10, 17). Nevertheless, after reaching menopause, women are also prone to abdominal visceral fat accumulation (17) and additionally have lower muscle mass than men. These sex-specific differences in body composition and distribution, combined with varying age-related physical and hormonal changes among women and men, might affect their physical functionality, rate of accumulation of various health deficits and overall health differently. We observed a strong positive association between high baseline FMI and VAT mass and the odds of pre-frailty/frailty later in life among women in our study population, but these associations were weaker and closer to unity in men. Very few men in our study sample had low grip strength (n = 5), indicating comparatively better muscle strength among the rest of the men. This might have had a protective effect against fat mass, resulting in a weaker non-significant association between FMI or VAT mass and frailty among men. However, caution should be applied while interpreting the results for men because the low number of men with certain frailty indicators in this specific sample limits the power to reach any conclusion.

It is challenging to establish the directionality of the association with cross-sectional studies, especially when it comes to body composition, physical function and frailty-related variables, because these might reinforce each other. So, the main strength of the present study is its prospective design with a follow-up of community-dwelling participants over 8 years. Furthermore, we were able to identify the pre-frail/frail population at baseline and exclude them, allowing us to avoid the problem of reverse causality. However, as information on walking speed was missing at baseline, those who had slow walking speed but no other frailty indicators might have been misclassified as robust. Also, we could not account for fluctuations in adiposity that might have occurred during the follow-up period. To address the limitations associated with adiposity assessed using traditional anthropometric measurements, we used DXA-derived adiposity measures. DXA measures have higher accuracy compared with widely used simple anthropometric measures, skinfold techniques and BIA. Furthermore, when compared with other more accurate tools such as CT and MRI, DXA is less resource-demanding, involves negligible radiation exposure and provides reliable measures of body composition, including VAT (16, 38). We used FMI as a measure of total adiposity, because it accounts for height, is not affected by fat-free mass and is considered to be more reliable compared with the widely used body fat percentage (37). We assessed frailty using a slightly modified version of Fried et al.'s frailty definition (4), which is one of the most widely used definitions of frailty (58). Although this focuses on declining physiological reserves and functions among older adults, it does not account for psychological, cognitive or specific nutritional aspects of frailty, which could be a limitation. In the present study, the objectively assessed frailty indicators, i.e., low grip strength and slow walking speed, aligned with Fried's definition, but the questionnaires used for assessing self-reported indicators, i.e., exhaustion, low physical activity and unintentional weight loss, differed slightly. Moreover, the pre-frail/frail population in this study is mainly pre-frail, with a score of 1, and we had to combine pre-frailty and frailty as a single outcome due to the low prevalence of frailty at follow-up. The self-reported frailty indicators might have resulted in the misclassification of robust participants as pre-frail or vice versa. Although it would have been interesting to examine the association of adiposity measures with pre-frailty and frailty separately, information on the identification of pre-frailty and the associated risk factors is highly relevant because it would allow the timely administration of effective intervention to delay or reverse the progression of frailty and prevent adverse outcomes (59). Last of all, our study is prone to selection bias, because the participants lost to follow-up were older and had slightly higher total and central adiposity measures and other risk factors for pre-frailty and frailty.

Globally, the proportion of older adults is on the rise and so is obesity. Excess adiposity during adulthood and at an older age may increase the risk of various health deficits that can contribute to different age-associated disorders, including frailty. Our study results indicate that higher fat mass and VAT mass during adulthood are associated with a higher likelihood of pre-frailty and frailty in later years among women, thus highlighting the significance of preventing adiposity, especially central adiposity, for healthy aging. The findings in men were more uncertain, and further longitudinal studies that include larger sample sizes, repeated measurements on advanced device-based measures, as well as frailty status, are required to fully understand the effect of excess adiposity on the development of frailty and the impact of a sex difference on this association.

## Funding

This work was supported by the Throne Holst Foundation (Grant number N/A). The project also received funding from Aktieselskabet Freia Chocolade Fabriks Medisinske fond (Grant number N/A). The funders had no role in the research manuscript's design, conduct, analysis, interpretation, or drafting. Open access funding provided by University of Oslo (incl Oslo University Hospital).

## Electronic Supplementary Material

Supplementary material, approximately 141 KB.
