# Supplementary material for: Dual-Energy X-Ray Absorptiometry Derived Adiposity Measures and Pre-Frailty/Frailty among Norwegian Adults: The Tromsø Study 2007–2015
Source: J Nutr Health Aging. 2023 May 25;27(6):403–10. doi: 10.1007/s12603-023-1920-2 (PMC12880043; doi:10.1007/s12603-023-1920-2)
Supplement: Supplementary file 1 — Supplementary material, approximately 141 KB. [file mmc1.doc]

**Supplementary materials**

**Supplementary Table 1 Comparison between Fried et al.’s suggested criteria for frailty and modified frailty indicators used in the present** study

| **Frailty** | **Fried et al. (4)** | **Tromsø6** | **Tromsø7** |
| --- | --- | --- | --- |
| **Exhaustion** | Questions from the Center for Epidemiologic Studies Depression Scale:  (a) I felt that everything I did was an effort  (b) I could not get going  How often in the last week did you feel this day?  0 = Rarely or none of the time (<1 day)  1 = Some or a little of the time (1–2 days)  2 = A moderate amount of time (3–4 days)  3 = Most of the time  **Exhausted: “A moderate amount of time (3–4 days)” or “Most of the time”** | Hopkins Symptom Checklist (HSCL-10):  During the last week, have you experienced that everything is a struggle?  1 = No complaint  2 = Little complaint  3 = Pretty much  4 = Very much  **Exhausted: “Pretty much” or “Very much”** | Hopkins Symptom Checklist (HSCL-10):  During the last week, have you experienced that everything is a struggle?  1 = No complaint  2 = Little complaint  3 = Pretty much  4 = Very much  **Exhausted: “Pretty much” or “Very much”** |
| **Physical activity** | Minnesota Leisure Time Activity Questionnaire asking about walking, chores (moderately strenuous), mowing the lawn, raking, gardening, hiking, jogging, biking, exercise, cycling, dancing, aerobics, bowling, golf, singles, tennis, racquetball, calisthenics, swimming  The kcal/week expended was calculated using a standardized algorithm. Lowest 20% were identified, resulting in following cut-off for frailty:  Men: <383 kcal of physical activity/week  Women: <270 kcal of physical activity/week | Describe your exercise and physical exertion in leisure time over the last year (Saltin–Grimby’s scale):  1 = Reading, watching TV/screen or other sedentary activity  2 = Walking, cycling, or other forms of exercise at least 4 hours a week  3 = Participation in recreational sports, heavy gardening, snow shoveling, etc. at least 4 hours a week  4 = Participation in hard training or sports competitions, regularly several times a week  **Low physical activity level: “Reading, watching TV/screen or other sedentary activity”** | Describe your exercise and physical exertion in leisure time over the last year (Saltin–Grimby’s scale):  1 = Reading, watching TV/screen or other sedentary activity  2 = Walking, cycling, or other forms of exercise at least 4 hours a week  3 = Participation in recreational sports, heavy gardening, snow shoveling, etc. at least 4 hours a week  4 = Participation in hard training or sports competitions, regularly several times a week  **Low physical activity level: “Reading, watching TV/screen or other sedentary activity”** |
| **Weight loss** | In the last year, have you lost more than 10 pounds (4.5 kg) unintentionally (not due to dieting or exercise)?  **Frail: “Yes”** | Have you involuntarily lost weight during the last 6 months? (Malnutrition Universal Screening Tool)  0 = No  1 = Yes  **Lost weight: “Yes”** | Have you involuntarily lost weight during the last 6 months? (Malnutrition Universal Screening Tool)  0 = No  1 = Yes  **Lost weight: “Yes”** |
| **Grip strength** | Measured by Jamar dynamometer (kg)  Maximal strength in dominant hand (3 trials)  Stratified by sex and BMI quartiles. Lowest 20% were identified, resulting in the following cut-off for frailty:  **Men Cut-off for grip strength (kg) criterion for frailty**  BMI <24 ≤29 kg  BMI 24.1–26 ≤30 kg  BMI 26.1–28 ≤30 kg  BMI >28 ≤32 kg  **Women**  BMI <23 ≤17 kg  BMI 23.1–26 ≤7.3 kg  BMI 26.1–29 ≤18 kg  BMI >29 ≤21 kg | Measured by Martins Vigorimeter (bar); Multiplied by 100 to convert it into kPa; Divided by the conversion factor obtained from the Jamar–Martin conversion table: For females Martin (balloon 3) to Jamar (notch 2): 2.43 and for males Martin (balloon 5) to Jamar (notch 2):1.68.  Stratified by sex and BMI quartiles as per Fried’s definition:  **Men Cut-off for grip strength (kg) criterion for frailty**  BMI <24 ≤29 kg  BMI 24.1–26 ≤30 kg  BMI 26.1–28 ≤30 kg  BMI >28 ≤32 kg  **Women**  BMI <23 ≤17 kg  BMI 23.1–26 ≤17.3 kg  BMI 26.1–29 ≤18 kg  BMI >29 ≤21 kg | Measured by Jamar dynamometer (kg); Strongest measurement from 3 trials in each hand  Stratified by sex and BMI quartiles as per Fried’s definition:  **Men Cut-off for grip strength (kg) criterion for frailty**  BMI <24 ≤29 kg  BMI 24.1–26 ≤30 kg  BMI 26.1–28 ≤30 kg  BMI >28 ≤32 kg  **Women**  BMI <23 ≤17 kg  BMI 23.1–26 ≤17.3 kg  BMI 26.1–29 ≤18 kg  BMI >29 ≤21 kg |
| **Walking speed** | Time to walk (seconds) 15 feet at usual pace stratified by sex and height (gender-specific cut-off at medium height): Lowest 20% were identified, resulting in the following cut-off for frailty:  **Men Cut-off for walking speed criterion for frailty**  Height ≤173 cm ≥7 s  Height >173 cm ≥6 s  **Women**  Height <159 cm ≥7 s  Height >159 cm ≥6 s | Not Available | SPPB: Short Physical Performance Battery – walking test  Fastest of two times (seconds) to walk 4 m stratified by sex and height according to Fried's gender-specific cut-off. Converted to feet from meters.  **Men Cut-off for walking speed criterion for frailty**  Height ≤173 cm ≥7 s  Height >173 cm ≥6 s  **Women**  Height <159 cm ≥7 s  Height >159 cm ≥6 s  **Only available in Tromsø7** |
| **Frailty status** | Frailty score:  0 = Robust  1–2 = Pre-frail  ≥3 = Frail | Frailty score:  0 = Robust  1–2 = Pre-frail  >3 = Frail  Pre-frailty/frailty score:  0 = Robust  >1 = Pre-frail/frail | Frailty score:  0 = Robust  1–2 = Pre-frail  >3 = Frail  Pre-frailty/frailty score:  0 = Robust  >1 = Pre-frail/frail |

**Supplementary Table 2 Longitudinal association between adiposity measures and frailty components: The Tromsø Study 2007–2015**

|  | **Women** | | | **Model 1** | **Model 2** |  | **Men** | | **Model 1** | **Model 2** |
| --- | --- | --- | --- | --- | --- | --- | --- | --- | --- | --- |
|  | **Normal** | **Low** | **OR (95% CI)** | | **OR (95% CI)** |  | **Normal** | **Low** | **OR (95% CI)** | **OR (95% CI)** |
| Grip strength | 179 | 14 |  | |  | Low grip strength | 106 | 5 |  |  |
| FMI (kg/m^2^) |  |  | 1.16 (0.99–1.35) | | 1.14 (0.97–1.34) | FMI (kg/m^2^) |  |  | 0.75 (0.45–1.24) | 0.56 (0.28–1.11) |
| VAT (g)^a^ |  |  | 1.07 (0.99–1.16) | | 1.07 (0.98–1.17) | VAT (g)^a^ |  |  | 0.95 (0.83–1.08) | 0.94 (0.82–1.07) |
| Walking speed | 173 | 20 |  | |  | Low walking speed | 102 | 9 |  |  |
| FMI (kg/m^2^) |  |  | 1.19 (1.04–1.37) | | 1.18 (1.02–1.36) | FMI (kg/m^2^) |  |  | 1.39 (1.05–1.85) | 1.57 (1.11–2.23) |
| VAT (g)^a^ |  |  | 1.06 (0.99–1.14) | | 1.06 (0.98–1.14) | VAT (g)^a^ |  |  | 1.10 (1.003–1.20) | 1.12 (1.01–1.25) |
| Physical activity | 186 | 18 |  | |  | Low physical activity | 120 | 17 |  |  |
| FMI (kg/m^2^) |  |  | 1.26 (1.09–1.47) | | 1.26 (1.08–1.49) | FMI (kg/m^2^) |  |  | 1.25 (1.02–1.54) | 1.43 (1.10–1.84) |
| VAT (g) ^a^ |  |  | 1.08 (1.01–1.16) | | 1.09 (1.01–1.18) | VAT (g)^a^ |  |  | 1.05 (0.99–1.12) | 1.06 (0.99–1.14) |
|  | **No** | **Yes** |  | |  |  | **No** | **Yes** |  |  |
| Exhaustion | 216 | 6 |  | |  | Exhaustion | 137 | 3 |  |  |
| FMI (kg/m^2^) |  |  | 1.03 (0.79–1.35) | | 1.03 (0.81–1.33) | FMI (kg/m^2^) |  |  | 0.87 (0.50–1.52) | 0.82 (0.44–1.51) |
| VAT (g)^a^ |  |  | 1.06 (0.95–1.20) | | 1.06 (0.93–1.19) | VAT (g)^a^ |  |  | 0.99 (0.84–1.16) | 0.98 (0.82–1.17) |
| Unintentional weight loss | 211 | 15 |  | |  | Unintentional weight loss | 123 | 15 |  |  |
| FMI (kg/m^2^) |  |  | 0.88 (0.73–1.05) | | 0.87 (0.72–1.05) | FMI (kg/m^2^) |  |  | 1.02 (0.81–1.29) | 0.98 (0.77–1.26) |
| VAT (g) ^a^ |  |  | 0.90 (0.80–1.02) | | 0.90 (0.79–1.02) | VAT (g)^a^ |  |  | 1.00 (0.93–1.07) | 0.98 (0.92–1.06) |
| Model 1: adjusted for age.  Model 2: adjusted for age, alcohol intake, smoking status and self-reported health at baseline,  ^a^ORs (95% CI) are per 100-g increase in VAT mass.  CI, confidence interval; FMI, fat mass index; OR, odds ratio; VAT, visceral adipose tissue. | | | | | | | | | | |

**Supplementary Table 3 Descriptive baseline characteristics of eligible Tromsø6 participants who attended Tromsø7 versus non-attenders: The Tromsø Study 2007–2016**

|  | **Frailty status** | |  |
| --- | --- | --- | --- |
|  | **Not attended Tromsø7**  ***n* =  166**  **(% (*n*))** | **Attended Tromsø7**  ***n* = 384**  **(% (*n*))** | ***P* value** |
| **Age, mean (SD)** | 72.6 (6.4) | 68.3 (5.3) | <0.01^a^ |
| **Married or Cohabiting** |  |  |  |
| Married/Cohabiting | 62.1 (103) | 76.8 (295) | <0.01 |
| Living alone | 37.9 (63) | 23.2 (89) |  |
| **Self-perceived health status** |  |  |  |
| Good | 52.7 (87) | 68.9 (262) | <0.01 |
| Poor | 47.3 (78) | 31.1 (118) |  |
| **Social Support** |  |  |  |
| Enough good friends | 86.8 (132) | 92 (335) | 0.06 |
| Not enough good friends | 13.2 (20) | 8 (29) |  |
| **Educational level** |  |  |  |
| Primary/Partly secondary | 46.3 (75) | 39.1 (149) |  |
| Upper secondary | 35.8 (58) | 31.2 (119) | 0.02 |
| College/University | 17.9 (29) | 29.7 (381) |  |
| **Smoking status** |  |  |  |
| Current smokers | 14.6 (23) | 12.3 (47) |  |
| Former smokers | 48.7 (77) | 50.1 (192) | 0.52 |
| Never | 36.7 (58) | 37.6 (144) |  |
| **Alcohol** |  |  |  |
| Never/Abstaining | 21.5 (35) | 15.1 (57) |  |
| Infrequent drinkers | 62.6 (102) | 63.1 (238) | 0.09 |
| Frequent drinkers | 15.9 (26) | 21.8 (82) |  |
| **Comorbidity** |  |  |  |
| No comorbidity | 60.8 (101) | 75.5 (290) | <0.01 |
| Comorbidity | 39.2 (65) | 24.5 (94) |  |
| **BMI, mean (SD)** | 27.0 (3.8) | 26.5 (3.8) | 0.25^a^ |
| **WC, mean (SD)** | 95.5 (10.9) | 93.9 (11.2) | 0.15^a^ |
| **FMI (kg/m^2^), mean (SD)** | 9.0 (3.2) | 8.5 (3.1) | 0.06^a^ |
| **VAT (g), mean (SD)** | 1164 (706) | 1077 (714) | 0.18^a^ |
| Values are percentage (number); *P* value: χ^2^ test for categorical variables. ^a^*P* value: Student’s *t*-test.  BMI, body mass index; FMI, fat mass index; SD, standard deviation; VAT, visceral adipose tissue; WC, Waist circumference. | | | |
